# Evaluating a Web-Based Coaching Program Using Electronic Health Records for Patients With Chronic Obstructive Pulmonary Disease in China: Randomized Controlled Trial

**DOI:** 10.2196/jmir.6743

**Published:** 2017-07-21

**Authors:** Lan Wang, Lin He, Yanxia Tao, Li Sun, Hong Zheng, Yashu Zheng, Yuehao Shen, Suyan Liu, Yue Zhao, Yaogang Wang

**Affiliations:** ^1^ Community Nursing Section School of Nursing Tianjin Medical University Tianjin China; ^2^ Internet Section Information Center Tianjin Medical University Tianjin China; ^3^ Health Service Management School of Public Health Tianjin Medical University Tianjin China; ^4^ Respiratory Unit Department of Respiratory Care Tianjin First Center Hospital Tianjin China; ^5^ Respiratory Unit Department of Respiratory Care General Hospital of Tianjin Medical University Tianjin China

**Keywords:** chronic obstructive pulmonary disease, electronic health records, Web-based coaching program

## Abstract

**Background:**

Chronic obstructive pulmonary disease (COPD) is now the fourth leading cause of death in the world, and it continues to increase in developing countries. The World Health Organization expects COPD to be the third most common cause of death in the world by 2020. Effective and continuous postdischarge care can help patients to maintain good health. The use of electronic health records (EHRs) as an element of community health care is new technology in China.

**Objective:**

The aim of this study was to develop and evaluate a Web-based coaching program using EHRs for physical function and health-related quality of life for patients with COPD in China.

**Methods:**

A randomized controlled trial was conducted from 2008 to 2015 at two hospitals. The control group received routine care and the intervention group received routine care with the addition of the Web-based coaching program using EHRs. These were used to manage patients’ demographic and clinical variables, publish relevant information, and have communication between patients and health care providers. Participants were not blinded to group assignment. The effects of the intervention were evaluated by lung function, including percent of forced expiratory volume in 1 second (FEV1%), percent of forced vital capacity (FVC%), peak expiratory flow (PEF), maximum midexpiratory flow; St George’s Respiratory Questionnaire (SGRQ); Modified Medical Research Council Dyspnea Scale (MMRC); and 6-Minute Walk Test (6MWT). Data were collected before the program, and at 1, 3, 6, and 12 months after the program.

**Results:**

Of the 130 participants, 120 (92.3%) completed the 12-month follow-up program. There were statistically significant differences in lung function (FEV1%: F1,4=5.47, *P*=.002; FVC%: F1,4=3.06, *P*=.02; PEF: F1,4=12.49, *P*<.001), the total score of SGRQ (F1,4=23.30, *P*<.001), symptoms of SGRQ (F1,4=12.38, *P*<.001), the activity of SGRQ (F1,4=8.35, *P*<.001), the impact of SGRQ (F1,4=12.26, *P*<.001), MMRC (F1,4=47.94, *P*<.001), and 6MWT (F1,4=35.54, *P*<.001) between the two groups with the variation of time tendency.

**Conclusions:**

The Web-based coaching program using EHRs in China appears to be useful for patients with COPD when they are discharged from hospital into the community. It promotes the sharing of patients’ medical information by hospital and community nurses, and achieves dynamic management and follow-up analysis for patients’ disease. In addition, this program can postpone the decreasing rate of lung function, improve quality of life, decrease dyspnea, and increase physical capacity.

## Introduction

Chronic obstructive pulmonary disease (COPD) is a major cause of morbidity and mortality, and is the fourth leading cause of death worldwide [1]. Because of the complex and progressive trajectory of COPD, interventions are needed that slow disease progression and prevent hospital readmissions [2]. However, longitudinal follow-up interventions can be costly, time consuming, and burdensome for health providers and study participants [3]. Computer-tailored intervention strategies have been shown to be more cost effective than standard care [4].

Electronic health records (EHRs) are scientific computerized systems that replace and expand on functions previously provided by paper medical records. EHRs can maintain and update millions of electronic records of patients and are easily transferable. They can also store, manage, and deliver information more efficiently than people can and permit multiple clinicians to simultaneously access the same patient records from different locations [5]. Beyond this, EHRs allow better communication between patients and clinicians. Using EHRs can expand the role of current health surveillance efforts and can help bridge the gap between public health practice and clinical medicine [6]. In addition, EHRs are a major component for current studies in health informatics, but different approaches should be applied [7].

In the United States, health care system EHRs have been widely adopted [8], and in primary care in the United Kingdom. Within these, there is a combination of diagnostic and therapy codes for COPD [9]. These EHRs play a beneficial role in the care of patients with chronic illness [10] and improve the quality and efficiency of health care [11]. In some developed countries, health care systems are encouraged to combine behavioral and medical health care and apply EHRs for health information exchange and quality improvement [12]. Various studies have examined the positive effect of using EHRs for ophthalmological patients [13,14].

In the United States, the implementation of EHRs has shown to improve the quality of life of patients in pulmonary rehabilitation [15], decrease medical error [16], reduce hospitalization costs [17,18], and contribute to the establishment of standardized evidence-based nursing. Moreover, EHRs have played an important role in the follow-up of patients with chronic illness and postpartum patients [19,20]. In 2010, the United States spent US $2.6 trillion on health care [21], and more than 76% of hospitals had adopted a basic EHR system by the end of 2014 [22]. In northern Europe, EHRs have been used widely and many clinics have adopted the clinical record and information system approach. This approach allows the combination of work on medical sciences and medical informatics and the improvement of the technical level of community hygiene and family medicine [23]. Studies on the prevalence of EHR use have not been completed in China.

Despite the widespread application of EHRs, policies to support health information exchange and to increase patients’ active participation still require improvement [24]. Of note, one qualitative study showed that EHRs were viewed as having adverse effects on physician workflow and team communication [25]. However, EHRs have many functions and include several kinds of data, and the aim to develop EHRs and the content of EHRs are the focus of research in the future [26].

Data for EHRs include personal medical records that consist of information related to the individual and family health history, results of physical examinations, immunization status, health care service use, and demographics. It has been suggested that placing this technology in patients’ homes may have an impact on patient involvement in self-monitoring, decision making, and self-care [27]. However, to our knowledge, no research has been published about clinical outcomes after using EHRs in a COPD setting. We developed EHRs based on the aim of our study, which involved the enrollment of health care providers and patients, enabling patients to access their own health records, exchange health information, and obtain a referral between tertiary and primary care. The main aim of this study is to develop EHRs as a hospital and community referral platform and to accomplish seamless nursing and transitional care. The secondary aim is to describe a prospective design to assess the feasibility and efficacy of implementing EHRs among patients with COPD in order to promote larger studies of the application of EHRs in China and the management of COPD in primary care.

## Methods

### Study Design

**Figure 1:**
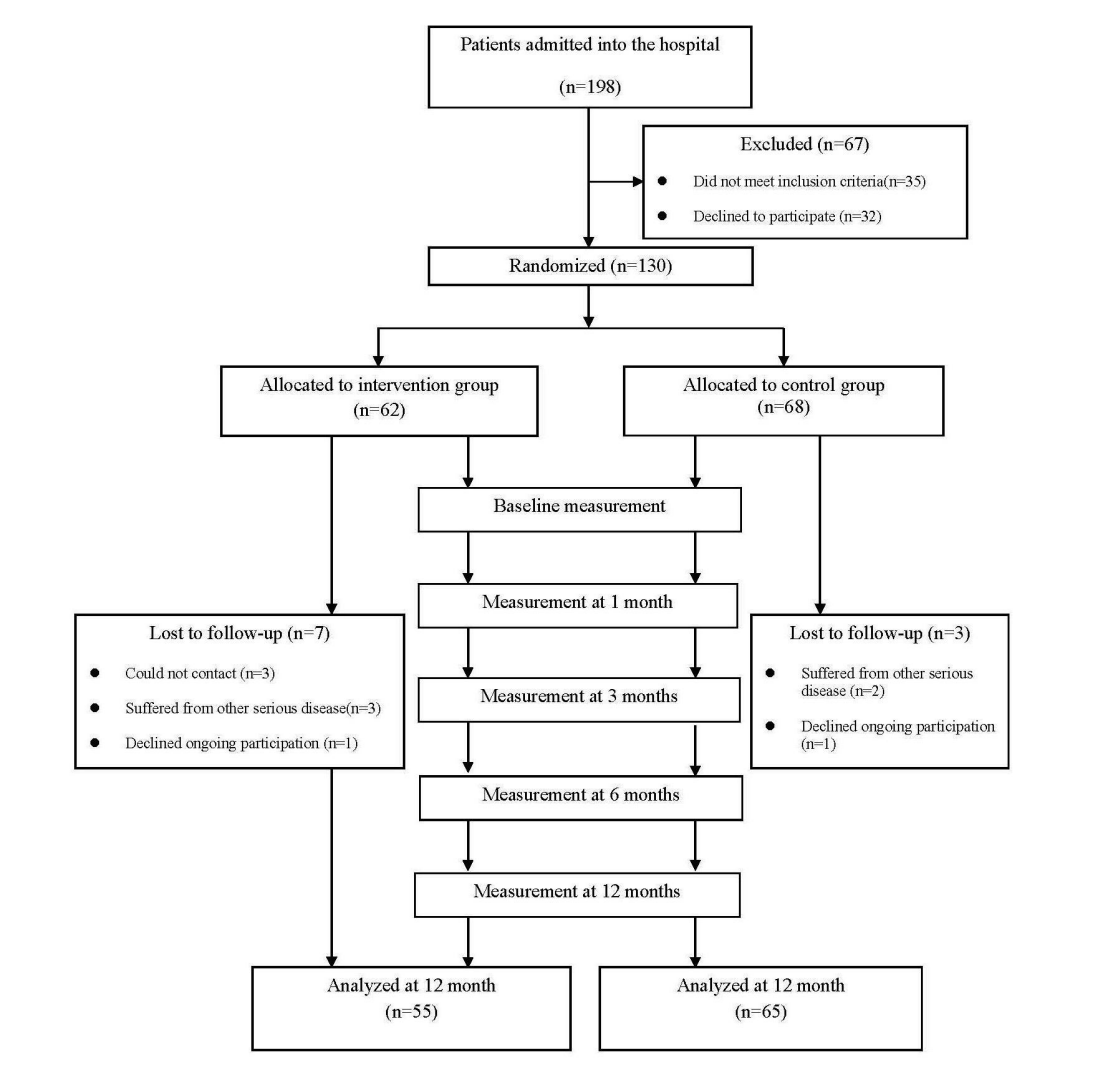
CONSORT flow diagram.

This was a randomized controlled trial, which compared an intervention group that received a Web-based coaching program using EHRs with a control group that did not receive the intervention after discharge from hospital. All participants received usual care before discharge, including medication guidance, lifestyle education, and regular reviews in order to help them manage their disease. Data were collected before the program and at 1, 3, 6, and 12 months after the program. Figure 1 illustrates the Consolidated Standards of Reporting Trials (CONSORT) flow diagram.

### Web-Based Coaching Program Using Electronic Health Records

The trial was developed by the research team, which included a clinical nurse, a head nurse, a community nurse, two respiratory physicians, and two nursing students (data collector). The clinical nurse taught disease-related information in the predischarge phase. The community nurse was responsible for the postdischarge follow-up using EHRs.

**Figure 2:**
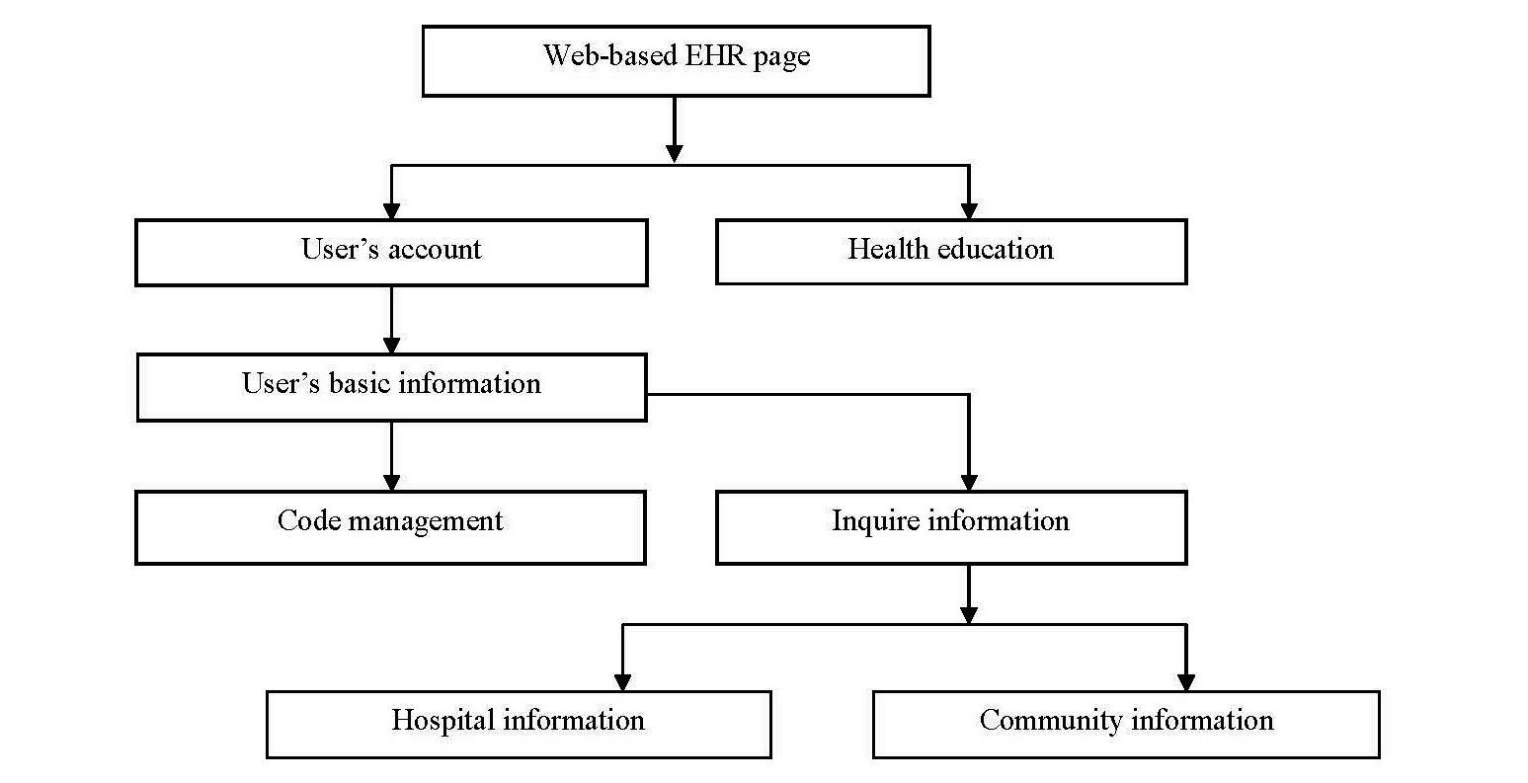
Participant log-in process.

**Figure 3:**
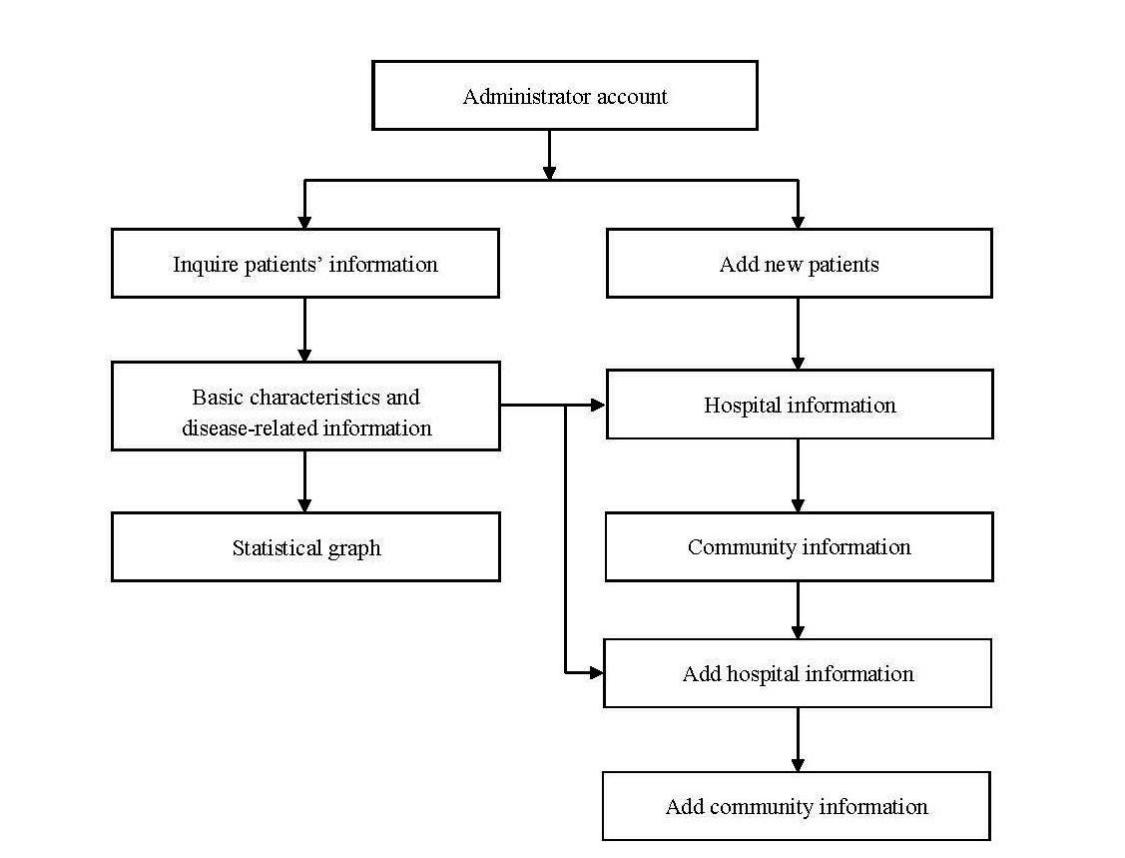
Administrator log-in process.

The EHRs were divided into two separate fields (Figures 2 and 3). There were two user spaces, one dedicated to the participants in the study (the front end) and the other one dedicated to the administrators of the platform (the back end). The patient-accessible personal health records were activated by the administrator logging into the system and inputting a username and code. Each participant received a unique identifier code generated by the system. The system was constructed to allow the input of the patient’s demographic information, record of admission, discharge, and community information. The system build included a statistical analysis function so that a curve graph could be produced of data variation that visually informed medical staff and patients of the trajectory of the disease. Once registered, the individual participant record was retrieved by entering the unique participant identifier. When the appropriate record was retrieved from the system, the administrator could add new information to the record. After the administrator assigned the username and code for the particular participant, the participant could access information about their disease and also health education content entered by the administrator, which now appeared in the participant field. The participant could connect with the community administrator through the EHR's system. This communication is similar to email function. Medical practitioners and nurses could write suggestions to the participants in the system and, when the patients logged in, they could view the message. Participants could also ask questions directed to the medical team using the same messaging function.

The implementation of the Web-based coaching program was based on sharing information within EHRs. All relevant data were recorded in the EHRs to allow the participants and administrators to refer to particular information at their convenience by Internet. There were two main information sets in these particular EHRs. One was related to health education and provided a resource for participants that included information about COPD and pulmonary rehabilitation instructions. The information related to COPD consisted of the cause of the disease, development, acute exacerbation, prognosis, medication information (name, route, dosage, and adverse reactions), oxygen therapy, and diet. Pulmonary rehabilitation instructions consisted of abdominal contraction and lip breathing, respiratory muscle exercise, aerobic exercise, and the importance of smoking cessation. The second information set was the participant’s particular management based on the progress of the disease. Tailored contents that match a user’s preference are more useful to motivate behavioral change [28].

The EHRs could store all the patients’ health information from hospital to community care. In this particular project, the individual also had control of their own records and managed their files. The records could be packaged and sent electronically. Because the participant had control of the records, they could supply their own health information to their health practitioners. Participants could read all their information and discuss it with the administrators by Internet within the EHRs.

The research team telephoned the participants every two weeks and made home follow-up visits at 1, 3, 6, and 12 months after discharge to promote adherence to the provided intervention methods and to collect data. The participants could telephone the community administrators when they had problems.

### Participants

Approximately 198 patients with COPD were asked to participate in the study from September 2008 to November 2014. A convenience sample of 130 participants agreed to participate and were recruited from two tertiary hospitals in Tianjin, China. The inclusion criteria were as follows: (1) a medically confirmed diagnosis of COPD based on the Chinese Medical Association Diagnostic Criteria, including percentage forced expiratory volume for 1 second (FEV_1_%)≤80% and forced expiratory volume for 1 second divided by forced vital capacity (FEV_1_/FVC)≤70%; (2) clear consciousness, able to speak Mandarin, and able to communicate; (3) discharged to a home where Internet and computer have been installed; and (4) able to be reached by telephone postdischarge. The exclusion criteria included the presence of comorbidities, such as allergic rhinitis, myocardial infarction, severe heart failure, and malignant tumor; living outside Tianjin; and no access to a computer and Internet at home.

The patients who consented to participate were assigned to the intervention or control group using a computer-generated randomized table. Permission to carry out the study was granted by Tianjin Medical University Ethics Committee. The study was conducted in accordance with the Declaration of Helsinki. All participants completed an informed consent form before entering the study.

### Data Collection

Patients who were definitively diagnosed with COPD were contacted the day after their admission to hospital. The questionnaires and clinical variables were collected the day before the patient’s discharge from the hospital and at 1, 3, 6, and 12 months after discharge.

### Measures

#### Demographics

The demographic variables included gender, age, education, career, history of cigarette smoking, whether it was the first admission, and disease classification.

#### Lung Function

A portable MicroLoop Spirometer (Jaeger, made in Germany) was used to test lung function. The index included FEV_1_%, the percent of forced vital capacity (FVC%), FEV_1_/FVC, peak expiratory flow (PEF), and maximal midexpiratory flow (MMEF_25%-75%_). These data were measured in the hospital ward using calibrated spirometers and the results were assessed in accordance with American Thoracic Society criteria. Participants were seated comfortably, the device was placed in the mouth, and the nose was blocked by hand to prevent nose ventilation. Then, the patient was instructed to make an explosive, fast, deep breath to generate the maximal expiratory flow volume (MEFV) curve; this was repeated three times [29].

#### St George’s Respiratory Questionnaire

St George’s Respiratory Questionnaire (SGRQ) was used as a respiratory disease-specific measurement of health status [30]. The SGRQ is a disease-specific instrument designed to measure the impact of respiratory symptoms on overall health, daily life, and perceived well-being. The instrument has shown good validity and reliability, with Cronbach alpha >.8. The questionnaire is divided into two sections. Section 1 includes frequency and severity of symptoms and section 2 covers activities that cause or are limited by breathlessness and impact social functioning and psychological well-being resulting from airway disease. Responses in the first section are given on a five-point Likert scale and in the second section as dichotomous variables (yes/no). Results are reported in four sections: symptoms, activities limited by breathlessness, psychosocial impact, and overall impact as a basis for the assessment of quality of life. Each section is presented as a weighted score. Scores range from 0 to 100, with higher scores indicating poorer health. A change of 4 units or more in the total score represents a clinically relevant change [30].

#### Modified Medical Research Council Dyspnea Scale

The degree of dyspnea was measured using the Modified Medical Research Council (MMRC) Dyspnea Scale [29]. This is a four-point visual analog scale: 0=dyspnea occurring only in intense activity, 1=dyspnea only when walking in a hurry or on ramp, 2=dyspnea identified by walking more slowly than others or needing pause for breath when moving at normal pace on flat ground, 3=dyspnea identified needing to pause for breath when walking on flat ground for 100 meters or several minutes, and 4=dyspnea identified as unable to leave the house or shortness of breath when dressing.

#### Six-Minute Walking Test

The 6-minute walking test (6MWT) has demonstrated validity and reliability to assess changes in functional capacity following pulmonary rehabilitation in patients with chronic obstructive lung disease [31]. Referring to the American Thoracic Society application guidelines, the researchers selected a 30-meter level and straight corridor in the ward marked on two sides. Patients started moving at normal speed from the start to the end point and returned, then repeated the process. The total time was 6 minutes. At the end of the 6MWT, the participant was asked to stop and the distance walked was recorded.

### Analysis

Data were analyzed using SPSS for Windows version 19.0 software (SPSS Inc, Chicago, IL, USA), with the level of statistical significance set at *P*<.05. We used descriptive statistics to analyze the participants’ characteristics. Repeated variant analysis was performed to test the data from the two groups collected predischarge and at 1, 3, 6, and12 months after discharge. Differences between the two groups were evaluated by *t* tests.

Expecting a difference between the groups at follow-up equivalent to an effect size of approximately 0.8, with a power of .80 and alpha at *P*=.05, the number of participants needed in each group was estimated to be 33.

During the intervention, seven of 62 participants (11%) in the study group dropped out (three changed addresses, two had cerebral infarction, one had a fracture, and one declined ongoing participation). Three of 68 participants (4%) in the control group were lost to follow-up (one had a cerebral infarction, one had myocardial infarction, and one declined ongoing participation). Therefore, 120 participants completed the study. The number of participants in the intervention group was 55 and in the control group was 65 (Figure 1).

### Participants’ Characteristics

The mean age of the 120 participants (47.5%, 58/120 men) was 70.6 (8.0) years. The severity of disease was evaluated as Global Initiative for Chronic Obstructive Lung Disease (GOLD) II (23.3%, 28/120), GOLD III (47.5%, 57/120), GOLD IV (29.2%, 35/120). There were no statistical differences between the intervention and control group in the main sociodemographic characteristics at baseline (Table 1).

#### Comparison of Lung Function Between the Two Groups

The variation of lung function in the intervention and control groups is described in Table 2.

**Table 1:** Comparison of demographics characteristics between two groups

Characteristics		Intervention group (n=55)	Control group (n=65)	χ^2^ (df)	*t* (df)	*P*
**Gender, n (%)**				3.5 (1)		.06
	Male	21(38%)	36(55%)			
	Female	34(62%)	29(45%)			
Age		69.33 (7.82)	71.85 (8.07)		–1.813 (1)	.07
**Education, n (%)**				0.5 (4)		.97
	Higher education	7(13%)	9(14%)			
	High school	8(15%)	8(12%)			
	Middle school	22(40%)	24(37%)			
	Primary school	17(31%)	22(34%)			
	None	1(1%)	2(3%)			
**Career, n (%)**				8.4 (3)		.08
	Worker	29(53%)	47(73%)			
	Manager	12(22%)	4(6%)			
	Officer	10(19%)	8(12%)			
	Farmer	2(3%)	4(6%)			
	None	2(3%)	2(3%)			
**Smoke, n (%)**				2.6(1)		.11
	Yes	21(38%)	16(25%)			
	No	34(62%)	49(75%)			
**First admission, n (%)**				1.1 (1)		.30
	Yes	28(51%)	27(41%)			
	No	27(49%)	38(59%)			
**Disease classification, n (%)^a^**				1.0 (3)		.23
	GOLD II	15(27%)	13(20%)			
	GOLD III	27(49%)	30(46%)			
	GOLD IV	13(24%)	22(34%)			

^a^ GOLD: Global Initiative for Chronic Obstructive Lung Disease; FEV_1_/FVC: forced expiratory volume in 1 second/forced vital capacity; GOLD II: FEV_1_/FVC <70%, 50% ≤FEV_1_<80% predicted; GOLD III: FEV_1_/FVC <70%, 30% ≤FEV_1_<50% predicted; and GOLD IV: FEV_1_/FVC <70%, FEV_1_<30% predicted.

**Table 2:** Variation tendency of lung function in the two groups.

Index of lung function^a^		*F*_2,4_	*P*
**FEV_1_%**			
	Time	11.235	.30
	Group	8.220	.01
	Time*group	5.474	.002
**FVC%**			
	Time	6.314	<.001
	Group	8.355	.01
	Time*group	3.055	.02
**FEV_1_/FVC**			
	Time	1.636	.17
	Group	0.430	.52
	Time*group	1.088	.37
**PEF**			
	Time	8.432	<.001
	Group	15.427	<.001
	Time*group	6.733	<.001
**MEF_75%_ FVC**			
	Time	1.393	.24
	Group	5.404	.02
	Time*group	2.621	.04
**MEF_50%_ FVC**			
	Time	0.145	.93
	Group	3.783	.05
	Time*group	2.701	.05
**MEF_25%_ FVC**			
	Time	5.172	<.001
	Group	2.039	.16
	Time*group	0.409	.77
**MMEF**			
	Time	3.754	.01
	Group	2.944	.09
	Time*group	1.241	.29

^a^ FEV_1_%: The percent of forced expiratory volume in 1 second in prediction; FEV_1_/FVC: forced expiratory volume in 1 second / forced vital capacity; FVC%: the percent of forced vital capacity in prediction; MEF_25%_ FVC, MEF_50%_ FVC, MEF_75%_ FVC: midexpiratory flow; MMEF: maximal midexpiratory flow; PEF: peak expiratory flow.

There was a tendency to change in FVC% (*P*<.001), PEF (*P*<.001), MEF_25%_ FVC (*P*<.001), MMEF (*P*=.01) in a year, and FEV_1_% (*P*=.01), FVC% (*P*=.01), PEF (*P*<.001), MEF_75%_ FVC (*P*=.02) were significantly different in the two groups. The intervention method and time had an interaction effect in FEV_1_% (*P*=.002), FVC% (*P*=.02), PEF (*P*<.001), and MEF_75%_ FVC (*P*=.04). The variation of variables is shown in Table 3.

**Table 3:** Comparison of lung function between the two groups before and after intervention with repeated data analysis of variance.

Item and group		Baseline	After intervention (month)				*F*_2,4_	*P*
			1	3	6	12		
**FEV_1_%**								
	Intervention, mean (SD)	0.38 (1.16)	0.41 (0.18)	0.41 (0.18)	0.41 (0.19)	0.42 (0.20)	2.324	.08
	Control, mean (SD)	0.33 (0.14)	0.33 (0.16)	0.32 (0.16)	0.31 (0.15)^b^	0.30 (0.15)^a,b,c,d^	4.863	.01
	*t*_1_	1.881	1.486	2.811	2.872	3.622		
	*P*	.06	.14	.01	.01	<.001		
**FVC%**								
	Intervention, mean (SD)	0.50 (0.17)	0.55 (0.18)^a^	0.56 (0.18)^a^	0.57 (0.18)^a^	0.56 (0.18)^a^	5.509	.001
	Control, mean (SD)	0.47 (0.19)	0.50 (0.16)^a^	0.48 (0.16)	0.47 (0.16)^b^	0.46 (0.14)^d^	3.533	.01
	*t*_1_	1.730	1.486	2.934	3.191	3.507		
	*P*	.32	.14	.004	.002	.001		
**PEF**								
	Intervention, mean (SD)	0.32 (0.16)	0.39 (0.19)^a^	0.41 (0.20)^a^	0.43 (0.20)^a,b^	0.41 (0.19)^a^	12.486	<.001
	Control, mean (SD)	0.28 (0.13)	0.27 (0.16)	0.29 (0.18)	0.29 (0.17)	0.27 (0.16)	0.686	.56
	*t*_1_	1.766	3.680	3.614	4.228	3.680		
	*P*	.08	<.001	<.001	<.001	<.001		
MEF_75%_ FVC								
	Intervention, mean (SD)	0.22 (0.17)	0.23 (0.19)	0.25 (0.21)	0.25 (0.19)	0.25 (0.19)	2.397	.06
	Control, mean (SD)	0.17 (0.13)	0.19 (0.17)	0.18 (0.17)	0.17 (0.15)	0.15 (0.12)	0.606	.18
	*t*_1_	1.669	1.341	2.103	2.468	3.369		
	*P*	.10	.18	.04	.01	.001		
**MEF_50%_ FVC**								
	Intervention, mean (SD)	0.18 (0.17)	0.19 (0.15)	0.19 (0.14)	0.19 (0.15)	0.21 (0.16)	1.809	.15
	Control, mean (SD)	0.15 (0.15)	0.14 (0.13)	0.15 (0.15)	0.14 (0.13)	0.13 (0.10)	0.932	.42
	*t*_1_	0.941	1.737	1.601	1.812	3.230		
	*P*	.35	.08	.11	.07	.002		
**MEF_25%_ FVC**								
	Intervention, mean (SD)	0.18 (0.15)	0.22 (0.17)^a^	0.23 (0.16)^a^	0.21 (0.15)^a^	0.22 (0.16)^a^	2.861	.04
	Control, mean (SD)	0.16 (0.13)	0.18 (0.13)	0.20 (0.18)^a^	0.17 (0.12)^c^	0.17 (0.12)	2.611	.048
	*t*_1_	0.865	1.388	0.913	1.418	1.789		
	*P*	.39	.17	.36	.16	.08		
**MMEF**								
	Intervention, mean (SD)	0.18 (0.15)	0.20 (0.16)	0.21 (0.15)^a^	0.20 (0.15)	0.22 (0.15)^a,d^	3.180	.03
	Control, mean (SD)	0.15 (0.12)	0.16 (0.14)	0.18 (0.17)	0.16 (0.13)	0.15 (0.12)	1.766	.16
	*t*_1_	1.275	1.395	1.187	1.577	2.621		
	*P*	.20	.17	.24	.12	.01		

^a^ Significant compared to baseline.

^b^ Significant compared to 1 month.

^c^ Significant compared to 3 months.

^d^ Significant compared to 6 months.

For FEV_1_%, there were no statistically significant differences in the intervention group (*P*=.08). However, there were significant differences in the control group (*P*=.01). From 3 months to 12 months, the intervention group had higher values than the control group (*P*=.01, *P*=.01, and *P*<.001, respectively).

For FVC%, there were statistically significant differences in the intervention group (*P*=.001) and the control group (*P*=.01). From 3 to 12 months, the intervention group had higher values than the control group (*P=*.004, *P=*.002, and *P=*.001, respectively).

There were statistically significant differences in the intervention group (*P*<.001) for PEF and no significant differences in the control group (*P*=.56). From 1 to 12 months, the intervention group had higher values than the control group (*P*<.001).

For maximal midexpiratory flow in MMEF_25%-75%,_ there were statistically significant differences in MEF_25%_ FVC between the intervention (*P*=.04) and control group (*P*=.048), and there were statistically significant differences in the intervention group (*P*=.03) for MMEF.

### Comparison of Quality of Life Between the Two Groups Before and After Intervention

The variation of SGRQ between the intervention and control group is described in Table 4.

There was a tendency to change in overall impact (*P*<.001), symptoms (*P*<.001), activities (*P*=.03), and psychosocial impact (*P*=.047) in a year. The intervention method and time showed an interaction effect in each variable. The variation of variables is shown in Table 5.

For overall impact, there were statistically significant differences in the intervention and control groups (*P*<.001). From 1 month to 12 months, the intervention group had higher values than the control group (*P=*.01, *P*<.001, *P*<.001, and *P*<.001, respectively).

There were statistically significant differences in symptoms in the intervention and control groups (*P*<.001). From 3 months to 12 months, the intervention group had lower values than the control group (*P=*.02, *P*<.001, and *P*<.001, respectively).

For activities, there were significant differences in the intervention group (*P<*.001). However, there were no significant differences in the control group. From 1 month to 12 months, the intervention group had lower values than control group (*P=*.002, *P=*.001, *P*<.001, and *P*<.001, respectively).

There were statistically significant differences in impact in the intervention (*P=*.02) and control groups (*P*<.001). From 1 month to 12 months, the intervention group had lower values than the control group (*P=*.03, *P*<.001, *P*<.001, and *P*<.001, respectively).

**Table 4:** Variation tendency on the St George’s Respiratory Questionnaire (SGRQ) in the two groups.

Item and group	Total		Symptom		Activity		Psychosocial impacts	
	*F*_1,4_	*P*	*F*_1,4_	*P*	*F*_1,4_	*P*	*F*_1,4_	*P*
Time	7.607	<.001	20.859	<.001	2.871	.03	2.477	.047
Group	21.894	<.001	8.781	.004	20.400	<.001	18.413	<.001
Time*group	23.300	<.001	12.384	<.001	8.351	<.001	12.259	<.001

**Table 5:** Comparison of quality of life between the two groups before and after intervention.

Item and group		Baseline	After intervention (month)				*F*_1,4_	*P*
			1	3	6	12		
**Total**								
	Intervention, mean (SD)	47.20 (18.73)	37.95 (20.89)^a^	35.02 (20.42)^a^	34.45 (19.95)^a^	31.35 (20.53)^a,b^	20.364	<.001
	Control, mean (SD)	51.22 (18.53)	47.95 (19.60)	51.03 (21.46)^b^	55.69 (20.90)^a,b,c^	57.92 (21.41)^a,b,c,d^	10.001	<.001
	*t*_1_	–1.177	–2.705	–4.163	–5.663	–6.904		
	*P*	.24	.01	<.001	<.001	<.001		
**Symptom**								
	Intervention, mean (SD)	61.87 (19.75)	41.04 (27.30)^a^	38.56 (24.71)^a^	38.84 (23.60)^a^	35.82 (21.32)^a^	16.194	<.001
	Control, mean (SD)	57.74 (19.77)	47.95 (19.60)^a^	49.95 (25.85)^a^	54.98 (23.48)^b,c^	58.03 (23.71)^b,c^	5.905	<.001
	*t*_1_	1.142	–1.355	–2.454	–3.745	–5.353		
	*P*	.26	.18	.02	<.001	<.001		
**Activity**								
	Intervention, mean (SD)	58.24 (23.16)	51.09 (24.38)^a^	50.13 (24.89)^a^	47.15 (25.43)^a^	43.96 (24.66)^a,b^	7.101	<.001
	Control, mean (SD)	65.49 (20.19)	64.58 (21.37)	65.02 (23.27)	68.77 (22.19)	69.00 (22.45)	1.996	.12
	*t*_1_	–1.834	–3.231	–3.382	–4.974	–5.818		
	*P*	.07	.002	.001	<.001	<.001		
**Impacts**								
	Intervention, mean (SD)	34.85 (21.01)	29.18 (20.31)^a^	26.00 (20.93)^a^	26.80 (21.04)^a^	22.89 (22.12)^a^	3.279	.02
	Control, mean (SD)	40.42 (20.10)	37.48 (20.97)	41.02 (22.43)^b^	44.49 (22.06)^b^	48.48 (22.88)^a,b,c,d^	7.851	<.001
	*t*_1_	–1.479	–2.191	–3.767	–4.470	–6.198		
	*P*	.14	.03	<.001	<.001	<.001		

^a^ Significant compared to baseline.

^b^ Significant compared to 1 month.

^c^ Significant compared to 3 months.

^d^ Significant compared to 6 months.

**Table 6:** Variation tendency of the Modified Medical Research Council (MMRC) Dyspnea Scale and 6-Minute Walk Test (6MWT) for the two groups.

Item and group	MMRC		6MWT	
	*F*_1,4_	*P*	*F*_1,4_	*P*
Time	21.090	<.001	0.394	.09
Group	56.522	<.001	9.631	.004
Time*group	47.940	<.001	35.541	<.001

### Comparison of Modified Medical Research Council Dyspnea Scale and Six-Minute Walk Test Between the Two Groups Before and After Intervention

The variation of the MMRC and 6MWT for the intervention and control groups are described in Table 6. There is a tendency to change in MMRC (*P*<.001) and 6MWT (*P*<.001) in a year. The intervention method and time had an interaction effect in MMRC. However, there was no significant difference in the intervention time in the 6MWT. The variation of MMRC and 6MWT are shown in Table 7.

For the MMRC, there were statistically significant differences in the intervention (*P=*.01) and control groups (*P=*.001). From 1 month to 12 months, the intervention group had lower values than the control group (*P*<.001).

The 6MWT was only tested at baseline, 6 months, and 12 months when the participants were required to be reexamined in the outpatient service. There were significant differences between the intervention (*P<*.001) and control groups (*P<*.001). From 6 months to 12 months, the intervention group had higher values than the control group (*P=*.002 and *P*<.001, respectively).

**Table 7:** Comparison of the Modified Medical Research Council (MMRC) Dyspnea Scale and 6-Minute Walk Test (6MWT) between the two groups before and after intervention.

Item and group		Baseline	After intervention (month)				*F*_1,4_	*P*
			1	3	6	12		
**MMRC**								
	Intervention, mean (SD)	2.27 (1.01)	1.49 (1.05)^a^	1.35 (1.09)^a^	1.22 (0.91)^a,b,c^	1.13 (0.98)^a,b,c,d^	36.306	.01
	Control, mean (SD)	2.54 (0.89)	2.63 (0.86)	2.62 (0.90)	12.71 (0.88)	2.86 (0.79)^a,b,c,d^	4.859	.003
	*t*_1_	1.537	6.537	6.996	8.626	10.728		
	*P*	.13	<.001	<.001	<.001	<.001		
**6MWT^e^**								
	Intervention, mean (SD)	267.40 (121.09)	—	—	286.63 (125.38)^a^	297.28 (113.22)^d^	12.932	<.001
	Control, mean (SD)	224.07 (102.31)	—	—	210.18 (101.20)^a^	198.89 (98.84)^a,d^	37.951	<.001
	*t*_1_	1.804			3.133	4.321		
	*P*	.07			.002	<.001		

^a^ Significant compared to baseline.

^b^ Significant compared to 1 month.

^c^ Significant compared to 3 months.

^d^ Significant compared to 6 months.

^e^ The 6MWT was only tested at baseline, 6 months, and 12 months when the participants were at the hospital because this test must be taken in the hospital.

## Discussion

### Principal Results

This study examined the effects of a Web-based coaching program using EHRs for patients with COPD. The intervention group had a statistically significant better pulmonary function measured by FVC% and PEF, physical capacity measured by 6MWT, health-related quality of life measured by SGRQ, and lower dyspnea degree measured by MMRC during the 1-year follow-up.

The EHRs are databases that health care providers use to record patient-related information to track care [32,33]. The effects of EHRs have been validated effectively in a pharmacogenetic study and also in a study of asthma exacerbation among school-aged children [34,35]. However, one qualitative study found that participants’ low technological issues resulted in low satisfaction with the technology [36]. Because of several factors associated with high or low compliance with Web-based interventions [37,38], we recommend the use of a combination of Internet and non-Internet engagement to enhance follow-up of all participants.

The EHRs have contributed to the health management of people with chronic illness. Provision of patient-centered advice including personal diet and alimentary advice and personal intervention methods based on gender, age, health, environment, employment, and medical service can make a difference. The EHRs in our study were a continuous, comprehensive, individualized, health information database. The system had a large capacity to store content, was low cost, occupied a small amount of computer memory, and enabled a convenient reference point related to COPD. Patients could navigate their own records at any time at home.

This study was consistent with other studies which demonstrated that comprehensive therapy methods, such as medication, oxygen, respiratory exercise, diet, and health education could not change the decline of FEV_1_% altogether. However, our program could postpone the decreasing speed of FEV_1_%. The FEV_1_% of the control group began to decline at the end of 3 months and patient quality of life also declined as identified by SGRQ. The rate of decline in FEV_1_% is representative of the natural history of COPD [39]. With the aggravation of airway obstruction, FEV_1_% has a declining trend and an irreversible pathological process. Although the decline of FEV_1_% could not change, a study by Garcia et al [40] demonstrated that respiratory muscle exercise could increase FVC%, which was a way to judge airway obstruction and had a relationship with trachea resistance and conformance of pulmonary tissue. Our study also showed that the Web-based EHR coaching program was correlated with an increase in FVC%. Our intervention taught patients how to make a complete expiration through a breathing technique that can increase FVC%.

The program improved the quality of life for patients with COPD compared to the control group, whose quality of life declined. The SGRQ, an index predicting quality of life with COPD, was an effective and sensitive score to evaluate quality of life with COPD. This result shows the opposite finding compared to a recent study on the long-term effects of an Internet-mediated pedometer-based walking program in the United States that only reported there were no significant differences between the intervention and control groups [41]. Therefore, the content in the program made a big impact on patient quality of life. Our study showed that although quality of life in the intervention group improved notably, the intervention could not reverse airway obstruction, only postpone the ingravescence rate of airway obstruction. Although the irreversibility of airway obstruction was closely related with quality of life, the SGRQ is not a good indicator of irreversibility, which is related to the mortality and frequency of acute exacerbation [42,43].

The intervention contributes to a decrease in the degree of dyspnea. This program includes Web-based EHRs containing considerable information regarding prevention, treatment, pulmonary rehabilitation, and disease variation, which could encourage patients to actively engage in healthy behaviors and increase adherence to medication, oxygen therapy, and respiratory exercise. Consequently, this may improve patient endurance by decreasing systematic oxidative stress and decrease the dyspnea level by decreasing oxygen consumption and physiological dead space [44].

In our study, the program was related to an extended 6-minute walking distance, which strengthened endurance and improved quality of life. Physical capacity refers to a person’s overall ability to function and “undertake the physically demanding activities of daily living” [45]. Relevant studies have tested that different types of interventions, such as telephone support, a mobile telephone program, computer-based program, or diary-recorded home-walking intervention, could increase physical capacity for patients with COPD compared to standard care [46-49]. However, one study showed that telephone mentoring for home-based walking demonstrated no benefit to the exercise capacity measured by the 6MWT [50]. Therefore, this Web-based coaching program using EHRs gives patients more confidence to control dyspnea and is recommended for use in the hospital, community, and home setting. During the study period, the researchers found that the 6MWT was not an instrument currently used in hospitals in China for patients with COPD and only the participants who were recruited in this study used this measure. We had hoped that respiratory physicians would use this instrument more regularly as a necessary examination that they must consider to assess patients’ adaptation to exercise.

### Limitations

Our study had several limitations. First, we were not able to evaluate selection bias because we could not differentiate between participants who enrolled versus those who declined participation. Second, many participants were elderly and unable to use EHRs by themselves, needing help from their family members. This condition may have limited the participants’ ability to navigate the EHRs when their families were not present. Third, we could not control the frequency of computer use by participants or coaching compliance, which could influence the effect of the intervention. Finally, our study setting was limited to Tianjin and did not include those with mild severity of COPD (GOLD I), limiting the generalizability of our results in all the patients with four disease classifications of COPD in China.

### Conclusion

The study established a Web-based coaching program using EHRs, which included Internet and non-Internet as a valuable component of intervention for COPD management in China. The program contributed to improved quality of life for people with COPD through better respiratory function and physical capacity. It also promoted hospital and community nurses sharing patients’ medical information, and achieved dynamic management and follow-up analysis.

## Appendix

Multimedia Appendix 1 CONSORT-EHEALTH checklist (V1.6).

## Abbreviations

COPD: chronic obstructive pulmonary disease
EHR: electronic health record
FEV1%: percent of forced expiratory volume in 1 second
FEV1/FVC: forced expiratory volume in 1 second/forced vital capacity
FVC%: percent of forced vital capacity
GOLD: Global Initiative for Chronic Obstructive Lung Disease
MMEF25%-75%: maximal midexpiratory flow
PEF: peak expiratory flow
